# Clinicians’ views of factors of importance for improving the rate of VBAC (vaginal birth after caesarean section): a study from countries with low VBAC rates

**DOI:** 10.1186/s12884-016-1144-0

**Published:** 2016-11-10

**Authors:** Ingela Lundgren, Patricia Healy, Margaret Carroll, Cecily Begley, Andrea Matterne, Mechthild M. Gross, Susanne Grylka-Baeschlin, Jane Nicoletti, Sandra Morano, Christina Nilsson, Joan Lalor

**Affiliations:** 1 Institute of Health and Care Sciences, Sahlgrenska Academy, University of Gothenburg, Box 457, SE-405 30 Gothenburg, Sweden; 2 School of Nursing and Midwifery, National University of Ireland, Upper Newcastle Road, Galway, Ireland; 3 School of Nursing and Midwifery, Trinity College Dublin, 24 D’Olier Street, Dublin 2, Ireland; 4 Midwifery Research and Education Unit, Hannover Medical School, Carl-Neuberg-Str. 1, 30625 Hannover, Germany; 5 Universita Degli Studi di Genova, Via Balbi 5, 16126 Genova, Italy; 6 IRCCS Azienda Ospedaliera Universitaria S. Martino IST, Largo R. Benzi, 10 16132 Genova, Italy

**Keywords:** VBAC, CS, Clinicians, Focus groups, Qualitative study, Content analysis, Midwifery

## Abstract

**Background:**

Caesarean section (CS) rates are increasing worldwide and the most common reason is repeat CS following previous CS. For most women a vaginal birth after a previous CS (VBAC) is a safe option. However, the rate of VBAC differs in an international perspective. Obtaining deeper knowledge of clinicians’ views on VBAC can help in understanding the factors of importance for increasing VBAC rates. Focus group interviews with clinicians and women in three countries with high VBAC rates (Finland, Sweden and the Netherlands) and three countries with low VBAC rates (Ireland, Italy and Germany) are part of “OptiBIRTH”, an ongoing research project. The study reported here aims to explore the views of clinicians from countries with low VBAC rates on factors of importance for improving VBAC rates.

**Methods:**

Focus group interviews were held in Ireland, Italy and Germany. In total 71 clinicians participated in nine focus group interviews. Five central questions about VBAC were used and interviews were analysed using content analysis. The analysis was performed in each country in the native language and then translated into English. All data were then analysed together and final categories were validated in each country.

**Results:**

The findings are presented in four main categories with several sub-categories: 1) “prameters for VBAC”, including the importance of the obstetric history, present obstetric factors, a positive attitude among those who are centrally involved, early follow-up after CS and antenatal classes; 2) “organisational support and resources for women undergoing a VBAC”, meaning a successful VBAC requires clinical expertise and resources during labour; 3) “fear as a key inhibitor of successful VBAC”, including understanding women’s fear of childbirth, clinicians’ fear of VBAC and the ways that clinicians’ fear can be transferred to women; and 4) “shared decision making – rapport, knowledge and confidence”, meaning ensuring consistent, realistic and unbiased information and developing trust within the clinician–woman relationship.

**Conclusions:**

The findings indicate that increasing the VBAC rate depends on organisational factors, the care offered during pregnancy and childbirth, the decision-making process and the strategies employed to reduce fear in all involved.

## Background

Caesarean section (CS) rates are increasing in both resource-intense and resource-poor countries [1]; however, of concern is the variation in CS rates internationally. For example, in Europe, the Netherlands, Slovenia, Finland, Sweden, Iceland and Norway have rates below 20 %, whereas Italy and Cyprus have national CS rates of 38 and 52 % respectively [2].

As intervention rates continue to rise, concerns have emerged regarding the associated procedure-related risks in terms of maternal morbidity and mortality [3, 4]. In the absence of a robust justification of these increasing rates, an exploration of clinicians’ attitudes to CS as a mode of birth is required [2, 5–7], since an understanding of the non-medical factors associated with the decision to perform a CS are becoming more important. These factors include maternal requests [8] and provider attitudes [9].

Also of interest is the variation in rates of the mode of birth such as elective repeat CS emergency CS in labour, and vaginal birth after previous CS (VBAC) following one previous CS. VBAC is an important mechanism for reducing the CS rate [10, 11] given that CS rates in nulliparous women are rising steadily. Based on a limited number of randomised trials comparing outcomes for women planning a repeat elective CS with those planning a vaginal birth [12], current evidence supports VBAC as a reasonable and safe option for most women [13]. VBAC is associated with a lower incidence of maternal mortality and a reduction in overall morbidities for mothers and babies [13]. Although evidence exists that for most women a VBAC is safe, practice varies significantly, with as few as 29–36 % of women in Ireland, Italy and Germany experiencing a VBAC compared with 45–55 % of women in Finland, Sweden and the Netherlands [14].

However, few studies about clinicians’ views of VBAC have been done. According to clinicians in countries with high VBAC rates, the important factors for improving the VBAC rate are related to the structure of the maternity care system in the country, the cooperation between midwives and obstetricians, and the care offered during pregnancy and birth [15].

Given the concern that exists regarding increasing CS rates worldwide, and the limited evidence available on clinicians’ views of VBAC, this study was designed to explore the views of clinicians from countries with low VBAC rates on factors of importance for improving VBAC rates.

## Methods

As part of the ongoing OptiBIRTH study, which aims to increase VBAC rates [16], an exploration of clinicians’ views of VBAC in three countries with low rates of VBAC was undertaken. A qualitative approach was employed, which is useful when little is known about the phenomenon of interest [17]. One method of collecting qualitative data is the focus group interview, which has its roots in social science and psychology. It is an efficient way to gather data from a group of individuals about their values and attitudes and the complex phenomena that originate from social interaction [18]. As the purpose of this phase of the study was to inform the development of an intervention to increase VBAC rates in countries with low rates, agreement was reached within the research team that the following five questions (which were generated by consensus) would be asked in the same order, in each site and in each country: What factors are important for VBAC? What are the barriers to VBAC? What is important to you as a professional? What are your views on shared decision making with women? How can women be supported to be confident with VBAC?

### Settings

Data were collected using focus group interviews in nine sites – three in Ireland, Germany and Italy respectively – from both rural and urban locations. Although the countries differ in some respects with regard to how maternity care is provided, there are many similarities. For example, all countries provide maternity care free at the point of use through the public health care system; however, private models of maternity care also run in parallel. The key features of note are that the publicly funded model of care is predominantly medically led and that the women give birth in a hospital setting. These factors are important when considering the national CS rate for any country, since significant variations in CS rates have been identified at the unit level, depending on whether the woman attends the public system or utilises health insurance to attend an obstetrician privately [5].

In each country, women following one CS are required to attend an antenatal appointment with a consultant obstetrician to discuss the options for birth in the hospital where the birth is planned to take place.

### Data collection and participants

Focus group interviews with clinicians were conducted during 2012–2013. In total, 71 clinicians participated; see Table 1. Clinicians were eligible for inclusion if they were involved in discussing VBAC and supporting women and their partners regarding the optimal mode of birth following previous CS. Clinicians who met the eligibility criteria – commonly midwives, obstetricians and neonatologists – were approached by researchers in each country (initials PH, AM, JN, MG), and if the clinicians expressed interest in participating in the study, they were offered an information leaflet and consent form. The focus group interviews were held at a local site for the convenience of the participants.

**Table 1:** Characteristics of participants

Ireland	FGI 1 (rural)	11 (4 midwives, 4 consultant obstetricians, 1 neonatologist, 2 non-consultant hospital doctors)
	FGI 2 (urban)	8 (4 midwives, 2 consultant obstetricians, 2 non-consultant hospital doctors)
	FGI 3 (rural)	12 (7 midwives, 3 consultant obstetricians, 2 non-consultant hospital doctors)
Italy	FG 1 (urban)	9 (4 midwives, 5 obstetricians)
	FG 2 (urban)	7 (5 midwives, 2 obstetricians)
	FG 3 (urban)	7 (3 midwives, 4 obstetricians)
Germany	FGI 1 (urban)	6 (2 midwives, 4 obstetricians)
	FGI 2 (urban)	3 (1 midwife, 2 obstetricians)
	FGI 3 (urban)	8 (5 midwives, 3 obstetricians)

FGI (Focus group interviews)

### Data analysis

When analysing the focus groups interviews, the authors were influenced by inductive conventional content analysis [19, 20]. In qualitative content analysis, the aim is to build a model to describe a phenomenon in a conceptual form, derived from the data [20]. Content analysis is a flexible, pragmatic method for developing and extending knowledge of the human experience of health and illness [19].

The focus group interviews were transcribed verbatim in the participants’ native language. The following steps were used during analysis: selecting the units of analysis; making sense of the data as whole; conducting open coding; using coding sheets; and grouping, categorising and abstracting the data [20]. The units of analysis were the sections of the interview texts that answered the five questions. Each participating researcher (PH, JL, AM, MG, JN, SM) in the three countries did open coding in their native language, resulting in 5–10 subcategories per question. In order to ensure consistency, two researchers in each country coded the data independently and clarified any remaining inconsistencies. At this point, each country forwarded their preliminary analysis in English to the first author (IL) and CN for the datasets to be synthesised. This synthesis allowed for similarities between countries to be identified and equally for context-specific findings to be noted for the implementation phase of the OptiBIRTH trial. Again, to ensure consistency and accuracy of interpretation (a critical step in forwards and backwards translation) the research team held several Skype meetings in English to discuss the coding and the findings. Rigour was maintained through “peer debriefing” and repeated validation of the findings by all team members as the data analysis proceeded and the findings emerged. In order to identify the quotations by country of origin, the following identifiers are added: Ireland (IR), Germany (G) and Italy (IT).

## Results

The findings are presented in four main categories: “parameters for VBAC”, “organisational support and resources for women undergoing a VBAC”, “fear as a key inhibitor of successful VBAC”, and “shared decision making – rapport, knowledge and confidence”. Each category contains a number of subcategories.

### Parameters for VBAC

Successful VBAC depends on several factors, not least a careful consideration of the previous obstetric history, the present obstetric factors, a positive attitude in all who are centrally involved, and strategies such as early follow-up after the first CS and antenatal classes*.*

#### The importance of the obstetric history

A key theme that emerged is that not all women are suitable candidates for VBAC – hence the importance of the obstetric history (in particular, progress in a previous labour) and consideration of potential risk factors in the selection process. Of interest, clinicians in Ireland and Italy considered obesity to be a factor that militates against offering a VBAC.

*A good history, I think, is very important, so that one really knows in preparation of the birth why the first was a CS, and discussions can take place at that point.* (G)

Clinicians in Ireland were also concerned as to whether the previous CS was planned or was an emergency procedure.

*If you look at the outcomes … the morbidity from an emergency CS is three times that of an elective one. So … there isn’t any massive benefit clinically in terms of reducing risk. Then you have the big risk of a very bad outcome [with a VBAC] hanging over you, which you don’t get with an elective CS.* (IR)

#### Present obstetric circumstances

Even when a VBAC is planned early in pregnancy, the plan is often reviewed again as the woman approaches term and, in particular, if the pregnancy extends beyond the expected date of delivery. Although opinions varied on whether women should or should not be offered an induction of labour once the pregnancy is prolonged, clinicians expressed increasing concern about the associated risk of uterine rupture. Clinicians highlighted that even when a decision to induce labour is made, a level of uncertainty exists as to the best time to undertake the procedure.

*I am happy to induce; are we happy to induce? I am in my own practice. I would prefer to induce them at T + 3 or 4 rather than let them go to T + 10 personally. … I look at these women who have had one previous CS as normal, so I don’t think about doing anything until they were postdates, as if they were normal.* (IR)

Although there was some discussion regarding the potential of a maternal request for VBAC, not all clinicians thought that the women should have an automatic right to choose their preferred option without consideration of the associated obstetric risks involved.

*I think that women shouldn’t have a right to choose a vaginal birth after CS. The decision should be the result of an overall evaluation, which can’t exclude vaginal birth. A process of assessment of suitability is necessary, leaving flexibility for the clinician.* (IT)

#### A positive attitude to VBAC in all who are centrally involved

Clinicians indicated that for VBAC to be successful, the woman must be motivated and willing to consider the options. Clinicians in Ireland were keen to stress that even when a woman has an open mind towards VBAC, the final decision on the mode of birth cannot be made until late in the pregnancy. Clinicians in Italy suggested careful evaluation of the woman’s suitability for VBAC is required. Clinicians in Ireland indicated they were positively disposed to supporting a woman to have a VBAC if they had laboured previously, and were enthusiastic about supporting these women to labour.

*At the first visit, I always put down are they open minded about it or are they keen for CS. And if they are keen for another CS, I put down: “Not un-keen on another CS”. … If they are open minded, you can play along with them, like if they come in spontaneous labour*. (IR)

Clinicians indicated that the impact of a negative attitude towards VBAC among their colleagues should not be underestimated as a potential barrier to increasing the rate of VBAC. This was particularly the case for those working alongside clinicians in private practice. In addition to hospital-based colleagues, clinicians in Ireland found the support, or lack thereof, from the family doctor, known as the GP (general practitioner), as crucial in achieving a successful VBAC.

*The GP is vital because there are some GPs who will send the women in and say: “She had a CS last time and I really feel she needs a CS this time” at 6 weeks of gestation. They are not always a barrier. There are some who are very supportive and some who are extremely negative. If the GP will support you, then you are in business*. (IR)

Supporting women to have a VBAC requires a positive attitude, good teamwork and sufficient experienced staff available to ensure success. Clinicians commented that the obstetric and midwifery staff must be convinced that it is possible for carefully selected women to give birth vaginally after a previous CS, and must cooperate with each other in supporting these women to achieve success. If this is not the case, then the woman may lose confidence in her ability to give birth vaginally.

*A woman was sure she wanted to give birth with a VBAC, but the obstetrician wanted her to sign an informed consent where he wrote that, despite his having explained all the risks of VBAC, the woman wanted to deliver vaginally and that he was available for CS any time during labour. The woman’s husband was shocked. After all this, the woman started saying: “Perhaps a CS would be better!” Everything went well, but the woman spent the whole time wondering if she was doing the right thing.* (IT)

The family and the social environment are also influential in the decision-making process. Women are influenced by family members and require a level of determination to achieve a VBAC. Hence, clinicians in Germany reported that it is important to know that the woman herself is motivated to achieve a VBAC.

*Yes, quite clearly also the motivation of the partner, the woman’s attending gynaecologist, the motivation of the midwife who leads the antenatal class, the motivation of female friends who have had a CS, who say that a spontaneous delivery was possible and somehow went well.* (G)

#### Early follow-up and antenatal classes

The topic of the potential for a VBAC in the future should be raised soon after the first CS birth (including information about why the CS was required), to “sow the seeds” and increase a woman’s confidence for giving birth vaginally next time.

*Well, actually, you would have to begin in prenatal care because that is when you have the first contact with the woman, perhaps even after the first CS. That you somehow make it clear to her that it does not mean that your second child also needs to come into the world by CS; you can also give birth naturally.* (G)

Clinicians in Ireland and Italy also commented that focused antenatal education classes, targeted at encouraging VBAC as an option, would offer the opportunity to provide women with consistent evidence-based information. It was suggested that these classes might include the participation of women who have already experienced a VBAC, either face to face or through sharing a recorded interview, in order to inspire the other women and reawaken a confidence in their potential to give birth vaginally.

### Organisational support and resources for women undergoing a VBAC

A successful VBAC requires clinical expertise and resources during labour.

#### VBAC requires clinical expertise

The staff must also have the requisite clinical experience in caring for women labouring after a previous caesarean section. Clinicians in Italy raised concerns that changes to obstetric training in the past decade have led to more clinicians choosing to sub-specialise in areas other than labour ward management, such as fertility and endoscopic surgery. Maintaining an appropriate level of competence in managing VBAC in a culture that favours sub-specialisation may be problematic in the future.

*Nobody can tell what will happen during a trial of labour (TOL), so we should say that a TOL is possible, but only if we have staff who are not overworked and exhausted*. (IT)

Obstetricians in Italy reported that in the past few decades, many have left the field of obstetrics in favour of other specialities with fewer unsocial working hours and higher remuneration packages. They indicated that lack of training due to the very low VBAC rates has an impact on clinical competence and consequently on the potential to increase VBAC rates.

*Nowadays we can see how the culture has affected the training of residents [junior obstetricians]. For residents, a previous CS means another CS. They have to be told that a woman can have a VBAC*. (IT)

However, clinicians in Italy emphasised that it is critical that VBACs are undertaken in a unit with expertise to support these women in labour. If that proficiency or experience is not available, then it is safer to repeat the CS.

*The patient shouldn’t get to a hospital where she’ll find a negative attitude to VBAC.* (IT)

#### VBAC requires resources during labour

Clinicians’ attitudes to and confidence in caring for women having a VBAC do vary, but appropriate staffing of birthing suites by those with relevant expertise was considered essential by the Irish and German clinicians.

*If you come on duty and you know you have someone who is having a trial of labour and there is another midwife who is very confident at that too, that is reassuring for you too. … And it goes back to staffing levels and to managers on the labour ward*. (IR)

In Ireland, it was suggested that specific expertise in managing VBAC is required. This could be achieved through the provision and staffing of a dedicated area to monitor these women in labour. In addition, such an area must have speedy access to an operating theatre in case a repeat CS is required.

*We need a place for the group of VBAC women, something between the labour ward and the antenatal ward.* (IR)

### Fear as a key inhibitor of successful VBAC

Understanding women’s fear of labour and vaginal birth is a key component in successful VBAC. Fear in clinicians may also be transferred to the women and may influence the outcome.

#### Understanding women’s fear of childbirth

Clinicians reported that fear of childbirth after a previously traumatic birth experience is a key component, and it is important to understand the basis of that fear when discussing VBAC with women.

*You have to think about what the fear is really about. Is the fear about pain or is the fear about having a labour, getting to 8 cm, getting stuck and then having an emergency section?* (IR)

A previous negative or traumatic birth experience is highly influential, and following up after the first birth is therefore critical. A previous negative childbirth experience with a long labour that ended up in an emergency CS was considered to be a barrier by clinicians in Italy. The clinicians in Germany stated that if women have a negative or traumatic first birth experience (for example, emergency CS or a baby born in poor condition), in an effort to avoid a repeat of this experience some women ask for an elective CS with the next pregnancy.

*I find the idea to reflect on the first birth quite good. …. If I know that the woman had a traumatic birth experience, I would tell her: “Listen, go home. I would like to see you in 6 weeks and again in 3 months.” Time enough to process the first birth. And when she is pregnant again, the issue must be revisited, simply to process it.* (G)

However, as a woman approaches term, clinicians indicated that, in their experience, the woman’s resolve may weaken as she acquiesces to outside influences such as family and information sources on the Internet.

*Sometimes it is not even us; it is not the mother. Sometimes it is the mother’s mother and her sister and all that out there [general agreement], and they come in with all the baggage into the clinic. They are all set up for a VBAC and they come into the clinic at 37 weeks freaking out, even though they are all set up for a VBAC and you are really in trouble then. It is very, very difficult to handle that “I am afraid, I am reading this”. And it is the Internet, it’s Dr. Google*. (IR)

#### Understanding clinicians’ fear of VBAC

The reassurance that VBAC is possible requires that the treating clinician also believes that this is the case and adopts an evidence-based approach to care planning and delivery. Despite a personal belief in the value of increasing VBAC rates, clinicians in Ireland and Italy also feared the consequences (personally) of a poor neonatal outcome.

*The medico-legal issues in Ireland are probably adverse compared with Sweden, where there is absolutely no chance of you being sued over a VBAC. … A high VBAC rate with a poor neonatal outcome is not acceptable. We live in a small community. … Your reputation is important. If you have a serious event, everyone knows and keeps talking about it for about 6 months … no one will give you a gold medal for a VBAC rate of 95 % if you make one mistake. It’s a cultural issue; the culture in Ireland is they [women in the community] keep talking and keep talking, and if the mother requests a planned CS, it’s very hard to refuse.* (IR)

Therefore, shared decision making between the clinician and the woman is a critical factor in achieving a VBAC, and having a fearful mother and a reluctant clinician will not bode well for success. If women are informed of the evidence indicating that VBAC is a safe option and are included in the decision, then it is harder for them to think of suing the obstetrician following an adverse outcome.

*Fear is very negative during labour. The obstetrician’s anxiety is transferred to the woman in labour, who hasn’t got the will she had before labour … after being in labour for a long time, the woman goes in the operating theatre and she hasn’t achieved her goal*. (IT)

#### Clinicians’ fear can be transferred to women

Clinicians in Germany indicated that their personal attitude to and motivation for VBAC are important, in both public and private practice settings. The clinicians in Germany were of the opinion that clinicians’ self-confidence is important because if the clinicians are confident, they will transfer this feeling to the women. Furthermore, if obstetricians are not authentic in their support for VBAC, the clinicians also believed that women sense that too.

*Whereas I do believe that they are sensitive to our personal attitudes. They are very sensitive and know: “She is quite confident” and “That is okay”, or if they themselves think: “Oh, they are all standing there”. That creates, I think, uncertainty. … And I think that it transfers quite quickly, before you know it yourself. Maybe a wrinkled nose; they already get the impression … before we are actually aware of it. So I think that our personal attitude is not to be underestimated as we approach the women. I believe that being genuine is still very important.* (G)

Clinicians in Ireland expressed concern as to who was fearful of VBAC – the woman or the clinician. Clinicians in Italy mentioned that clinicians should aim to control their anxiety. If not, midwives, for instance, may be called on to manage “triple anxiety”: their own, the obstetrician’s and the woman’s.

*A midwife is the link between the woman and the doctor, and if [the midwife] often normally is a little bit anxious, you can imagine if the woman has had a previous CS. The anxiety of the midwife is double; the obstetrician will enter the room and ask: “Is there progress? Only 1 cm?” It is a kind of anxiety that is difficult to manage: it is difficult to work impartially while dealing with the woman’s anxiety, the obstetrician’s anxiety and your own anxiety!* (IT)

### Shared decision making between women and clinicians – rapport, knowledge and confidence

Shared decision making requires consistent, realistic and unbiased information, and trust within the clinician–woman relationship.

#### Providing consistent, realistic and unbiased information

For women to make an informed choice, the information they receive must be factually correct and readily accessible. While all the clinicians agreed that women should be made aware that VBAC is an option, it is also important to address the risks and to highlight that a repeat CS is also a potential outcome.

*These women must be informed about everything – what being in labour involves after a CS, what is involved in a repeat CS – because it wouldn’t be fair if we only talked about the risks [of VBAC] and not about what will happen with a repeat CS.* (IT)

However, clinicians in Ireland thought that having faith in her obstetrician was highly influential in the woman’s decision-making process.

*The presence of her own personal obstetrician [is important]. I think it is an issue certainly with the small number of patients who are private … they want to know that you are going to be there. I think if you are transferring a patient to your colleague and they have only met you during the visits: “Oh look, just do a CS on the Thursday before you go on holidays”. … I think the barrier is the uncertainty about who is going to be looking after them*. (IR)

#### Trust within the clinician–woman relationship

Clinicians in Ireland and Germany suggested that giving information early in the pregnancy helps to build a woman’s confidence that she can achieve a VBAC. It can also help her to view VBAC as the “norm”, which is vital.

*Not many of them will make a decision at the first point of contact. They will want to go home and have a think about it. If we don’t start the discussion at the booking. … The idea is to have the decision taken before 36 weeks.* (IR)

Consequently, a relationship between the woman and the clinician that is based on trust is important to success, and a high level of continuity of carer is essential if this relationship is to be maintained. In the Italian context, a woman who would like a VBAC should be looked after by either her obstetrician or a pro-VBAC obstetrician when she is in labour. If the obstetrician in charge on the day is not pro-VBAC, the likelihood of success is diminished.

*Continuity of care is of fundamental importance. If a colleague and I believe in VBAC, when a woman wants to have a VBAC, we have to be on duty when that woman is in labour; otherwise, it will be a total failure.* (IT)

Therefore, when continuity of carer is not feasible, the clinicians suggested that a plan for the birth needs to be clearly documented in the woman’s case notes.

*It is very important that the plan that is made between woman and clinician is documented because of different people [on duty], different consultants, different registrars … as we do not cover the labour ward over 24 hours with the same person/consultant.* (IR)

There was much debate within the focus groups as to whether the woman should ultimately have the choice to request a VBAC or indeed a repeat CS given the risks associated with both options. Ultimately there was agreement that a shared decision was in the best interest of all concerned. It was also suggested that midwives and partners should be part of the process, to maximise support for the woman in labour.

## Discussion

### Strengths and limitations

Using a qualitative approach can result in a deep understanding of the phenomenon being explored. This method is preferable when little is known beforehand about the topic [20]. The qualitative approach selected meant that we were able to generate a dataset across three countries with a range of professionals for comparison. However, when seeking depth, the researcher often has to sacrifice on achieving a large sample size, thereby limiting the generalisability of the findings. As for all qualitative studies, the findings must be interpreted in relation to the study’s context [21]. To facilitate transferability to other contexts, the researcher should clearly describe the context, selection and characteristics of the participants, the method or methods of data collection and the process of analysis [20, 21], which we sought to do.

A limitation with a focus group is that some participants may be invisible as a result of others wielding more influence in the group. In contrast, individual interviews permit all participants to take part in the same way [18]. However, in this study, since the participants had volunteered to take part because of their interest in the subject, all of them actively contributed to the discussion.

### Interpretation

Parameters for consideration for VBAC include a careful consideration of the previous obstetric history and present obstetric circumstances, both factors identified as being of critical importance. Clinicians acknowledged that not all women are suitable for VBAC, and in certain circumstances such as limited VBAC experience among available staff, the option to undertake a repeat elective CS is preferred. The extent to which maternal requests for CS for non-medical reasons impact on CS rates is a contentious issue [22], with comparison across studies posing a challenge owing to the wide variations in what is understood by the term “maternal request” [23]. Clinicians indicated that maternal requests for elective CS were often due to a lack of robust information on VBAC combined with a woman’s fear of childbirth. The solutions suggested by clinicians are to address the knowledge deficit, to instigate routine debriefing around the previous birth and to encourage women to keep an open mind around the mode of birth. Clinicians spoke about the value of evidence in making decisions about the mode of birth, but were very clear that they took into account many other factors in their decision-making process. Studies have found that significant variations in the rates of attempted VBAC [24] suggest that the decision-making process around the birth mode after a previous CS is complicated and multidimensional for both women and care providers, and this certainly was the case for the clinicians in this study. According to a meta-synthesis of the views of women, they need evidence-based information on both the risks and the positive aspects of VBAC [25]. Some of the findings in our study highlight that an understanding of the impact of personal opinion and the consequent variation in practice is critical for clinicians when interpreting CS and VBAC rates, since this understanding is likely to influence the guidance women receive when exploring their options for birth.

A number of the clinicians indicated that the decision-making process should begin immediately following the first CS. Without some discussion around the birth and the necessity for the CS, women may develop a fear of childbirth and may be more likely to request a CS in a subsequent pregnancy. However, evidence to support the practice of early processing of previous birth experiences is yet to be generated. Previous traumatic birth experiences are related to future fear of childbirth [26, 27], and clinicians in this study spoke of the need to understand this fear by offering women opportunities to tell their birth story; in this way, the women have a way to share, understand and integrate their fears, concerns or feelings of disappointment, and missing pieces of information [28].

Our findings highlight that organisational support and resources for women undergoing VBAC are of importance, including both the clinical expertise and the resources for monitoring these women during labour. Definitions of one-to-one support in labour differ [29], but continuous support in labour has been shown to reduce CS and instrumental vaginal births [30]. Clinicians in this study cautioned that women labouring with a previously scarred uterus have a unique set of risks and therefore do require close one-to-one support and supervision in labour. Previous studies agree with the general consensus of opinion among participating clinicians in this study that women planning a VBAC should be cared for in a suitably staffed and equipped delivery suite, with readily available recourse to facilities for a CS and neonatal resuscitation should the need arise in line with professional guidance [31–33]. However, some clinicians reported that due to sub-specialisation in obstetric training, the availability of expertise in this area of labour management is in decline.

According to the present study, fear around VBAC may not just be an issue for women, but may also be a concern for clinicians. A study showed that the likelihood of undergoing a VBAC was increased in women cared for by obstetricians with low levels of anxiety [9]. Dahlen [34] emphasised that an important part of professional competence is achieving the balance between a fear of complications and a faith in the birthing process. Our data indicate that part of maintaining that balance is establishing who is fearful of VBAC (the woman or the clinician) and why. Such understanding could be promoted in maternity units by giving clinicians the time and the opportunity for mutual reflection on their clinical practice and for debriefing after adverse events.

Fear of litigation and increasing risk aversion have become common issues affecting clinical decision making [3, 35]. The participants spoke repeatedly about safety as a central issue around the mode of birth choices after previous CS, without perhaps appreciating fully all the dangers of repeat CS [13]. Participating clinicians would only support increasing VBAC rates if the risks of maternal and neonatal morbidity were acceptable in their eyes.

Good relationships and shared decision making between women and clinicians were vital when making decisions around VBAC. Women have consistently cited care providers as having significant external influence on the decisions they make during pregnancy [36, 37]. However, there is a growing body of evidence to suggest that clinicians and women conceptualise and interpret risk differently, with each bringing their own experiences and biases to the discussion [38–41]. Goodall et al. [40] have described similar variations between what women want to know and what health professionals believe that they should know. These variations can lead to challenges, with the lack of concordance between the expectations and preferences of women and those of clinicians potentially impacting negatively on building a trusting relationship.

Women require evidence-based information about the positive aspects of VBAC and their body’s ability to birth normally [42] if they are to be supported in making truly informed decisions regarding the mode of birth following CS. Clinicians should be sensitive to the fact that in addition to appraising hard data based on risk, women are frequently influenced by the obstetrician’s personal values, attitudes, experiences and expectations of birth when making decisions. Women are also influenced by the relationships they have with friends, family and other sources of maternity information, and the impact of these influences on the decision-making process should not be underestimated.

## Conclusion

According to clinicians in low VBAC countries, in seeking to improve the VBAC rate, careful consideration of the parameters for VBAC is of importance. A careful obstetric history, a positive attitude by all centrally involved and strategies such as early follow-up after the first CS require attention. If VBAC rates are to increase, organisational support and resources for women undergoing VBAC, including clinical expertise and resources during labour, are central to achieving a successful outcome. Fear is a key inhibitor of successful VBAC; therefore, understanding both women’s and clinicians’ fear is critical. Shared decision making requires the availability of consistent, realistic and unbiased information, as well as a trusting relationship between the woman and her clinician. Some of these findings are in line with a similar study with clinicians in high VBAC countries [15] – for example, trust in the clinician–woman relationship, a positive attitude of all centrally involved, early follow-up and fear reduction [15]. However, according to the study from the high VBAC countries, adopting a common approach, ensuring good cooperation between midwives and obstetricians, having the final decision on the mode of delivery made by obstetricians while still involving women, and strengthening women’s trust in VBAC are aspects that promote VBAC [15], but these aspects were not mentioned by the clinicians in the present study. These factors indicate a major difference between the views and attitudes of clinicians in countries with low VBAC rates and the views and attitudes of clinicians in high VBAC countries, a difference that would warrant consideration.
